# Salivary Tests: A New Personalized Approach for the Early Diagnosis of Oral and Periodontal Diseases

**DOI:** 10.3390/jpm12101636

**Published:** 2022-10-02

**Authors:** Gaetano Isola

**Affiliations:** Department of General Surgery and Medical Surgery Specialties, School of Dentistry, University of Catania, 95123 Catania, Italy; gaetano.isola@unict.it

Periodontitis is a chronic bacterial infection of the periodontium that results in the destruction of bone and connective tissue [1]. The main objectives of periodontal treatment should be early diagnosis and management, as it is difficult to rebuild the periodontium after alveolar bone loss. However, periodontitis frequently progresses without exhibiting any symptoms, and many patients do not seek out expert dental care until the periodontal damage has reached an irreversible stage. Furthermore, time-consuming clinical measurements are necessary for the current periodontitis diagnosis that could also control the early release of inflammatory mediators during periodontitis or after dental surgery [2,3]. The demand for near-patient testing to identify periodontitis remains unfulfilled. Saliva is the best biological fluid to use as a periodontitis near-patient diagnostic tool [4].

Saliva-based periodontitis diagnostic tests are now technically possible, according to recent advancements in point-of-care (POC) testing. There have been reports of a variety of promising salivary biomarkers linked to periodontitis. Based on the etiology of periodontitis, a panel of ideal biomarkers needs to be carefully chosen. The procedure for validating periodontitis POC diagnosis in a wide, heterogeneous patient group may present the most significant challenge [5].

One of the best biological fluids to use as a periodontitis diagnostic tool is saliva. Saliva can be taken repeatedly with no discomfort to the patient because saliva collection is easy, safe, and non-invasive. Saliva has already been found to include various interesting biomarkers correlated with periodontitis clinical characteristics. Locally produced proteins, genetic/genomic indicators such as DNA and mRNA, as well as a number of metabolites from both the host and the bacteria are all present in saliva. The detection of disease activity at each specific tooth location is not possible with saliva-based periodontitis diagnosis; this can only be achieved with standard clinical assessments. In this regard, saliva-based periodontitis diagnosis must be carried out as a form of point-of-care (POC) testing [6,7].

POC testing, also known as point-of-care testing, is medical testing carried out outside of a laboratory or close to the location of patient care, such as the patient’s bedside, the doctor’s office, or their home. Patients may easily identify their periodontitis at home and visit dental clinics at the appropriate time if periodontitis is detected by a POC device utilizing saliva. Current disease activity and treatment response can be conveniently tracked at the chairside in dental clinics. Due to the fact that periodontitis is linked to numerous systemic disorders, including atherosclerosis, coronary heart disease, diabetes mellitus, and rheumatoid arthritis, a POC device for diagnosing periodontitis would also help doctors determine the periodontal health of their patients.

In order to prevent the development of MRONJ, a typical side effect of medication used in conjunction with tooth extraction, doctors can take their patients’ periodontal health into account before prescribing bisphosphonates or other medications linked to MRONJ. Saliva testing for the diagnosis of periodontitis is now theoretically possible as a result of recent advancements in POC testing.

Biomarker signals in biofluids can now be found thanks to considerable advancements in technology. For instance, real-time monitoring of biomarkers in a small body fluid volume at POC sites is made possible by integrating microfluidic and lab-on-a-chip technologies. Lab-on-a-chip techniques combine various processing steps into a single tiny device, including sampling, sample preparation, detection, and data analysis. Blood, saliva, nasal aspirate, and urine are just a few of the clinical samples that can be analyzed by microfluidics-based equipment [8,9].

Soft tissues are damaged when periodontitis worsens, releasing enzymes and proteins that are involved in tissue deterioration into the saliva. MMP-8, MMP-9, HGF, lactate dehydrogenase, aspartate aminotransferase, and TIMP-2 are among the biomarkers of periodontitis that are particularly or potentially potent. Additionally, a recent metabolomic analysis of saliva found elevated levels of metabolites derived from macromolecular degradation in periodontitis, including uridine (DNA/RNA), lysolipids, oligo/mono-saccharides, fatty acids, and monoacylglycerol (glycerophospholipid and triacylglycerol) [10,11].

Bone damage in periodontitis can be detected using salivary biomarkers of bone remodeling. These include calcium, RANKL, osteonectin, and alkaline phosphatase. It has also been established that CAL and salivary calcium levels are positively correlated [12,13].

The diagnosis of periodontal and peri-implant diseases is based mostly on clinical assessments of pocket depth, attachment loss, and bleeding on probing and radiographic evaluation, as described in the Primer. These diagnostic techniques can only evaluate tissue loss that has already occurred; they cannot tell how the disease is progressing or how it will do so in the future. The episodic advancement of the disease course makes it more difficult to measure disease progression using standard clinical and radiological methods accurately. In addition to being present in gingival crevicular fluid, peri-implant sulcular fluid, mouthwash, and saliva, neutrophil collagenase, also known as matrix metalloproteinase 8 (MMP8), has been identified as a major collagenolytic enzyme that damages periodontal tissue in periodontitis and peri-implantitis. The pathogenic rise in MMP8 levels and their activation in oral fluids, which was not extensively explored in the Primer, are a critical indicator of active periodontal disease [14,15,16].

In fact, a quantitative POC-activated MMP8 (aMMP8) oral fluid test was repeatedly and independently validated in Finland, Germany, Nigeria, Turkey, the Netherlands, and the United States to successfully susceptible screen sites and patients, differentiate active and inactive periodontal sites, predict future disease progression, and monitor treatment response and maintenance therapy. As the test is positive (that is, the level of aMMP8 in oral fluids is high) prior to the onset of active periodontal disease, its predictive usefulness resides in its capacity to identify subclinical periodontitis before clinical or X-ray symptoms. The test can also detect early periodontitis in adolescents who are genetically prone to it. The aMMP8 test is therefore reliable in populations of both adolescents and adults. As a result, the test is excellent for determining the best preventative and therapeutic approaches and tracking the progression of the disease [17,18].

Saliva-based POC periodontitis diagnosis relies on candidate biomarker validation in large populations that appropriately consider variety, such as race, geography, gender, and age. In periodontal research, periodontitis has been diagnosed by examining the entire mouth or only a few teeth, and other criteria have also been employed to categorize the severity of periodontitis. Integration of the findings from many studies is challenging due to the wide variances in patient categorization. Additionally, differences in target biomarkers, detection techniques for salivary biomarkers, and salivary sampling techniques (e.g., resting vs. stimulatory; whole vs. individual gland sampling) hinder direct comparisons of data from other studies or sites.
